# Matrix-derived inflammatory mediator *N*-acetyl proline-glycine-proline is neurotoxic and upregulated in brain after ischemic stroke

**DOI:** 10.1186/s12974-015-0428-z

**Published:** 2015-11-21

**Authors:** Jeff W. Hill, Edwin M. Nemoto

**Affiliations:** Department of Neurosurgery, University of New Mexico Health Sciences Center, Albuquerque, NM 87131 USA

**Keywords:** *N*-acetyl proline-glycine-proline, Chemokine, CXCR2, Stroke

## Abstract

**Background:**

*N*-acetyl proline-glycine-proline (ac-PGP) is a matrix-derived chemokine produced through the proteolytic destruction of collagen by matrix metalloproteinases (MMPs). While upregulation and activation of MMPs and concomitant degradation of the extracellular matrix are known to be associated with neurological injury in ischemic stroke, the production of ac-PGP in stroke brain and its effects on neurons have not been investigated.

**Findings:**

We examined the effects of ac-PGP on primary cortical neurons and found that it binds neuronal CXCR2 receptors, activates extracellular signal-regulated kinase 1/2 (ERK1/2), and induces apoptosis associated with caspase-3 cleavage in a dose-dependent manner. After transient ischemic stroke in rats, ac-PGP was significantly upregulated in infarcted brain tissue.

**Conclusions:**

The production of ac-PGP in brain in ischemia/reperfusion injury and its propensity to induce apoptosis in neurons may link MMP-mediated destruction of the extracellular matrix and opening of the blood-brain barrier to progressive neurodegeneration associated with the initiation and propagation of inflammation. Ac-PGP may be a novel neurotoxic inflammatory mediator involved in sustained inflammation and neurodegeneration in stroke and other neurological disorders associated with activation of MMPs.

## Introduction

Chemokines and chemoattractant peptides direct leukocytes to sites of inflammation. The CXCR2 chemokine receptor is the primary receptor mediating neutrophil chemotaxis, and CXCR2 and its ligands, CXCL1 (growth-related oncogene α (GRO-α)), CXCL2 (growth-related oncogene β (GRO-β)), and CXCL8 (interleukin-8 (IL-8)) are implicated in injury processes in stroke and other neurological disorders. A role for the CXCR2 receptor in ischemia/reperfusion injury is suggested by upregulation of CXCR2 and its ligands in stroke and a reduction in infarction with CXCR2 antagonists [1–4]. CXCR2-mediated infiltration of neutrophils into reperfused tissue has been demonstrated to be a primary mediator of tissue injury in numerous ischemia/reperfusion models, including ischemic stroke [4], and neuroprotection associated with CXCR2 receptor blockade has been presumed to occur largely indirectly through attenuation of leukocyte migration and activation.

Several studies have demonstrated neuroprotection by inhibitors of matrix metalloproteinases (MMPs) in both animal and in vitro stroke models [5–8]. MMPs are believed to promote neuronal death via several mechanisms. Degradation of the extracellular matrix (ECM) by MMPs facilitates opening of the blood-brain barrier and promotes hemorrhagic transformation and infiltration of inflammatory cells to the ischemic site [9]. Loss of neuronal contact with the ECM following MMP activation may cause neuronal death by anoikis following loss of the contact-dependent neuronal survival signal [10]. Further, intracellular MMP-2 and MMP-9 are complicit in neuronal apoptosis in both in vitro and in vivo stroke models [11]. Neuroprotection by MMP inhibitors has been proposed to result from inhibition of the aforementioned processes.

In lung inflammatory disorders, such as cystic fibrosis (CF), persistent inflammation and neutrophilia promote tissue damage. Recent studies have identified elevated levels of MMP-9 in lung of CF patients. Interestingly, elevated levels of MMP-9 in CF lung correlate with levels of a novel matrix-derived molecule produced by the proteolytic cleavage of collagen [12]. This proline-glycine-proline tripeptide becomes N-acetylated (ac-PGP) and structurally mimics the CXCR2 receptor binding motif present in pro-inflammatory chemokines such as IL-8 [13]. Ac-PGP has been shown to be a potent inducer of neutrophil chemotaxis and stimulates neutrophils to release MMP-9 and IL-8 [12, 14]. Thus, the production of ac-PGP at sites of inflammation may promote tissue injury through a forward-feeding inflammatory cycle that persists after the initial insult. With these observations and the established role of MMP-9 in neurological injury in stroke, we hypothesized that ac-PGP could be produced in brain during ischemia/reperfusion injury. To investigate the potential involvement of ac-PGP in neuronal injury and inflammation in stroke, we characterized the effects of ac-PGP on primary cortical neurons and examined production of the molecule in brain and plasma after ischemic stroke in animals.

## Methods

### Peptide synthesis

*N*-acetyl proline-glycine-proline (ac-PGP) (072–57) and *N*-acetyl proline-glycine-glycine (ac-PGG) (custom synthesis) were synthesized, HPLC purified, and verified by mass spectrometry by Phoenix Pharmaceuticals.

### Primary neuron culture

Primary cortical neurons were prepared from Sprague-Dawley rat embryos on day 18 of gestation as described previously [11], with the following modifications. Neurons were plated in Neurobasal medium (Gibco) containing 4 % fetal bovine serum (FBS). Twenty-four hours after plating, half of the medium was replaced with Neurobasal medium containing 2 % B27 (Gibco) and 4 μM cytosine arabinoside. On day 5, half of the medium was replaced with Neurobasal medium containing 2 % B27. Experiments were performed in quadruplicate on day 7. Replicates were performed using neurons obtained from different animals. Two-hour oxygen-glucose deprivation (OGD) in glucose-free deoxygenated Hank’s buffered saline solution (HBSS) with 24-h reoxygenation was performed as described previously [11]. Control cells were similarly incubated in normal HBSS [11]. Unused cell culture medium was identical in composition to that present on day 7 of neuronal cultures (Neurobasal, 1 % FBS, 1.5 % B27). Cell extracts were prepared by sonication of cells in phosphate-buffered saline (PBS) containing HALT protease and phosphatase inhibitor cocktail (Thermo Scientific) and centrifugation at 20,000×*g* for 10 min. Primary neurons were treated with ac-PGP and ac-PGG peptides at the indicated doses or PBS vehicle. Measurement of apoptosis by terminal deoxynucleotidyl transferase-mediated dUTP nick-end labeling (TUNEL) assay, cleaved caspase-3 immunocytochemistry, and Western blots were performed as described previously [11]. Extracellular signal-regulated kinase 1/2 (ERK1/2) and caspase-3 antibodies were obtained from Cell Signaling Technologies, and SB225002 and anti-CXCR2 antibody (sc-32089) were obtained from Sigma-Aldrich and Santa Cruz Biotechnology, respectively.

### Middle cerebral artery occlusion

All animal procedures were approved by the University of New Mexico Institutional Animal Care and Use Committee and were in compliance with federal guidelines. Animals were anesthetized with 2 % isoflurane inhalant in 100 % oxygen during all surgical procedures. Ninety-minute middle cerebral artery occlusion (MCAO) was performed in 290–300 g male spontaneously hypertensive rats as described previously [15]. Infarction and survival rates were 100 % in MCAO animals. Blood plasma and brain tissue extracts were prepared 24 h after reperfusion onset. Citrate plasma was prepared from blood obtained by cardiac puncture at sacrifice using 1.8-ml citrate plasma collection tubes (Becton-Dickinson). Animals were transcardially perfused with 300 ml of ice-cold PBS, and brain tissue extracts (20 % tissue) were prepared by sonication of cortical tissue obtained within the zone of infarction (2 mm rostral to the bregma and 3 mm lateral to the midline) in PBS containing HALT protease and phosphatase inhibitor cocktail and centrifugation at 20,000×*g* for 10 min. Infarction was verified by 2,3,5-triphenyltetrazolium chloride (TTC) staining [15] of a coronal brain section adjacent to the section used to prepare infarcted cortical tissue extract. Sham extracts were similarly prepared from brain tissue in animals undergoing the MCAO procedure without the insertion of an occluding suture.

### LC-MS analysis of ac-PGP

Measurement of ac-PGP in cell culture medium, cell extracts, plasma, and brain tissue extracts by liquid chromatography-mass spectrometry (LC-MS) was performed by Alliance Pharma. Chromatographic separation was performed on a Symmetry C18 column (100 mm × 2.1 mm, 3.5 μm) (Waters). Formic acid (0.1 % in water) was used as mobile phase A, and 0.1 % formic acid in acetonitrile was used as mobile phase B. The chromatographic analysis was conducted using gradient elution as follows: 10 % B (0–0.1 min), from 10 to 90 % B (0.1–1.5 min), 90 % B (1.5–2.5 min), from 90 to 10 % B (2.5–2.6 min), and 10 % B (2.6–4.0 min). Column temperature was set at 25 °C. Sample injection volume was 25 μl, and separation was performed at a flow rate of 0.5 ml/min. An API 4000 triple quadrupole mass spectrometer (Sciex) with TurboIonSpray interface was operated in positive ionization mode with multiple reaction monitoring. Precursor to product ion transitions were 312.3–112.0 for ac-PGP and 329.2–162.2 for labetalol (internal standard). Sciex Analyst software was used for data acquisition and analysis. The calibration curve (analyte peak area/internal standard peak area versus analyte concentration) was obtained based upon the least squares linear regression fit (*y* = *mx* + *b*) with a weighting factor of 1/*x*^2^. The coefficient of determination (*r*^2^) was > 0.99.

### Statistical analysis

One-way analysis of variance (ANOVA) with Bonferroni’s multiple comparison tests was used to establish the statistical significance of comparisons between three or more treatment groups. Comparisons of two groups were performed using a two-tailed Student’s *t* test. All data are shown as means with standard deviation. Statistical significance was taken as a *p* value < 0.05 with a 95 % confidence interval. Statistical analyses were performed using GraphPad Prism software.

## Results

### Ac-PGP induces apoptosis in neurons through the CXCR2 chemokine receptor

**Fig. 1:**
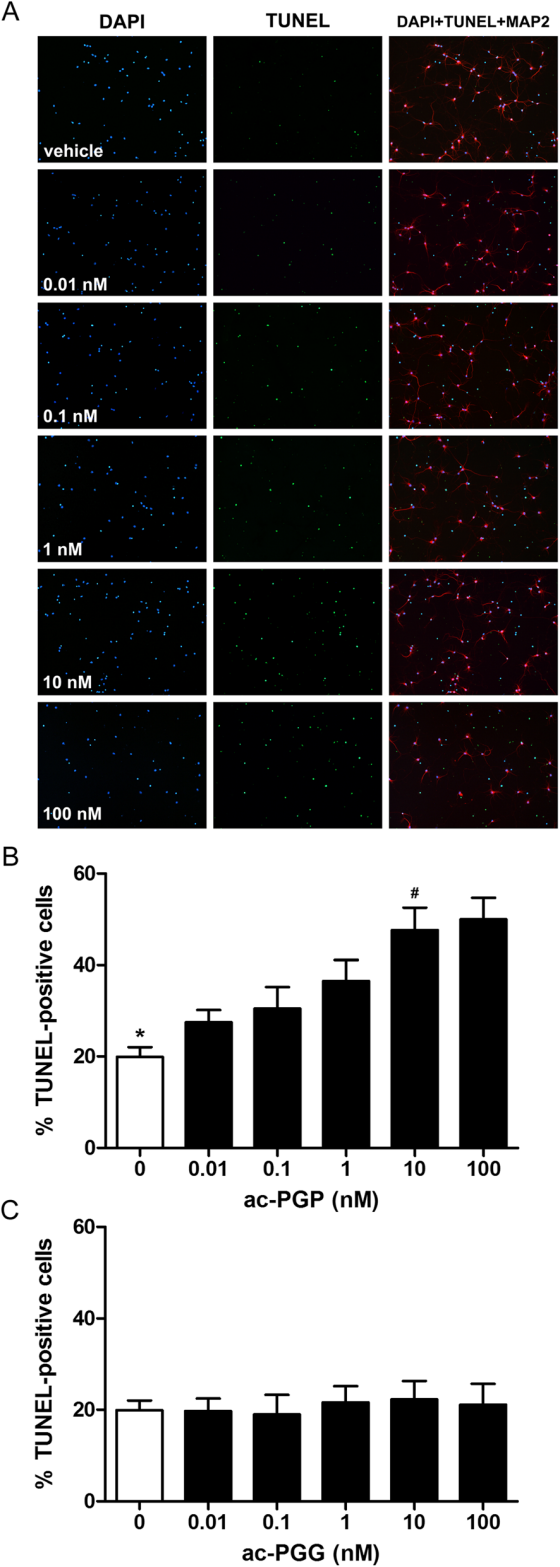
Ac-PGP induces apoptosis in primary cortical neurons. **a** Apoptosis in vehicle- and ac-PGP-treated neurons was measured by TUNEL assay 24 h after treatment. Nuclei were stained with 4',6-diamidino-2-phenylindole (DAPI). Neurons are indicated by microtubule-associated protein 2 (MAP2) staining. **b** Graphical representation of actual data shown in **a**. A statistically significant difference in apoptosis was found between vehicle and 0.1, 1, 10, and 100 nM ac-PGP (*p < 0.05 to *p* < 0.0001) and between 10 nM and 0.01, 0.1, and 1 nM ac-PGP (^#^ *p* < 0.0001 to *p* < 0.05). **c** A control ac-PGG peptide did not induce significant apoptosis in neurons at doses up to 100 nM

**Fig. 2:**
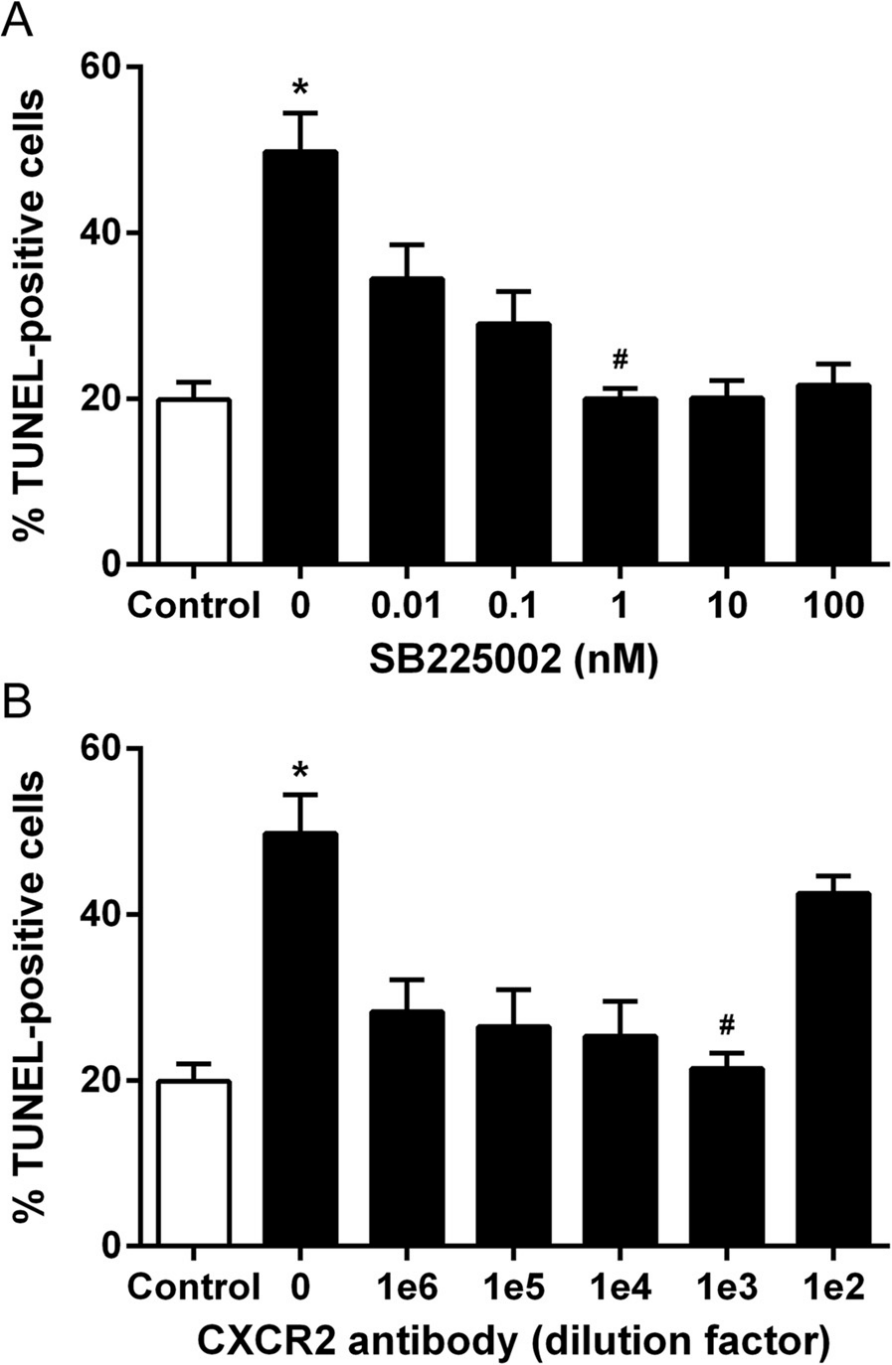
CXCR2 antagonist SB225002 and CXCR2 antibody protect neurons against ac-PGP-induced apoptosis. **a** Cells exposed to 10 nM ac-PGP for 24 h were first pretreated with SB225002 or anti-CXCR2 ligand-binding domain antibody for 30 min at the indicated dose. Cells pretreated with all doses of SB225002 were significantly less apoptotic than cells treated with ac-PGP only (*p < 0.0001). Cells pretreated with 1 nM SB225002 were significantly less apoptotic than cells treated with 0.01 and 0.1 nM SB225002 (^#^ *p =* 0.0001 to *p* < 0.05). **b** Cells pretreated with CXCR2 antibody at a 1:1000 (1e3) or greater dilution exhibited significantly less apoptosis than cells treated with ac-PGP alone (*p < 0.0001) while a 1e3 dilution produced significantly less apoptosis than a 1e2 dilution (^#^ *p* < 0.0001)

**Fig. 3:**
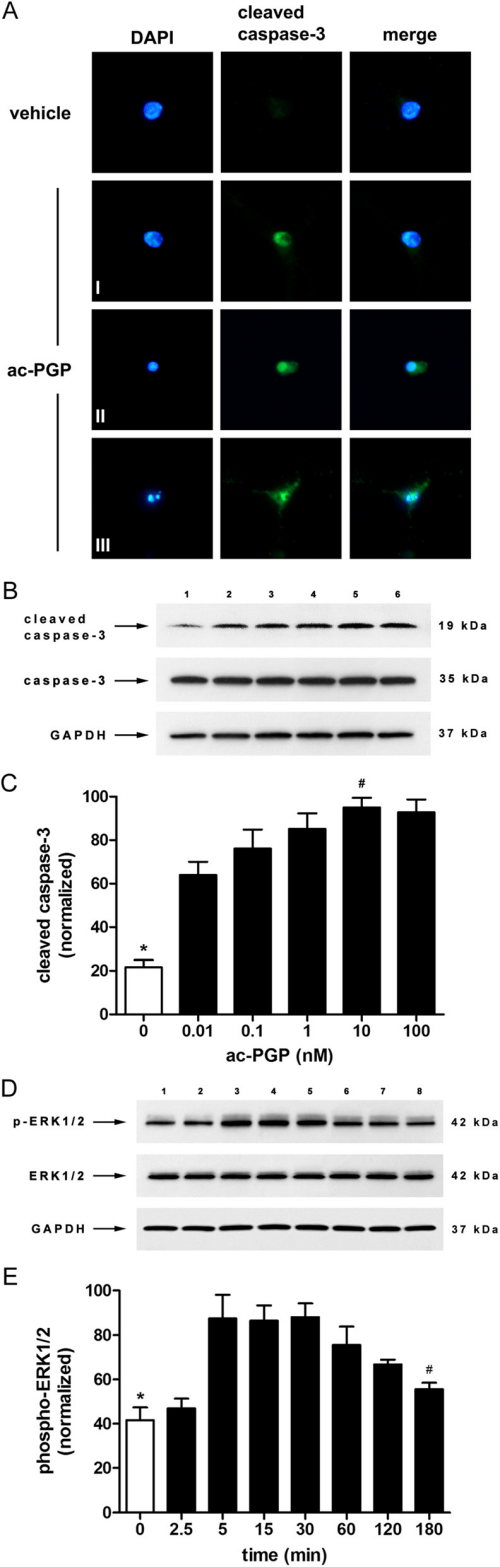
Caspase-3 cleavage and activation of ERK1/2 MAP kinase in cortical neurons exposed to ac-PGP. **a** Immunocytochemistry of neurons exposed to vehicle or 10 nM ac-PGP for 24 h. Cleaved caspase-3 was present at low levels in vehicle-treated cells and was significantly elevated in ac-PGP-treated cells. Stages of apoptosis are indicated I–III. *I* appearance of cleaved caspase-3 in the nucleus, *II* nuclear condensation, *III* nuclear fragmentation. **b** Western blot of cleaved caspase-3 in neuronal cell extracts after exposure to 0, 0.01, 0.1, 1, 10, or 100 nM ac-PGP for 24 h, lanes 1–6, respectively. **c** Quantitation of Western blot data shown in **b**. A statistically significant difference in cleaved caspase-3 levels between vehicle and all doses of ac-PGP was observed (*p < 0.0001), while 10 nM ac-PGP produced a significantly higher level of caspase-3 cleavage than 0.01 and 0.1 nM ac-PGP (^#^ *p* < 0.001 to *p* < 0.05). Cleaved caspase-3 levels were normalized to full-length caspase-3. GAPDH, cytoplasmic marker glyceraldehyde 3-phosphate dehydrogenase. **d** Western blot of phospho-ERK1/2 MAP kinase after exposure to 10 nM ac-PGP for the times indicated in **e**. Phospho-ERK1/2 levels were normalized to total ERK1/2. A statistically significant difference in ERK1/2 phosphorylation was observed between neurons treated with vehicle and ac-PGP for 5, 15, 30, and 60 min (*p < 0.005 to *p* < 0.05). ERK1/2 phosphorylation was significantly decreased at 180 min compared to 30 min (^#^ *p* < 0.05)

In primary neurons, ac-PGP induced apoptosis in a dose-dependent manner (Fig. 1). Control ac-PGG peptide was non-toxic at equivalent concentrations. Blockade of the CXCR2 receptor with a specific CXCR2 antagonist or anti-CXCR2 antibody inhibited ac-PGP-induced neuronal apoptosis (Fig. 2). The induction of apoptosis in neurons was confirmed by the measurement of caspase-3 cleavage following exposure to ac-PGP (Fig. 3a-c). Prolonged activation of ERK1/2 mitogen-activated protein (MAP) kinase was observed in neurons treated with ac-PGP (Fig. 3d, e).

### Ac-PGP is produced in primary neuron cultures and is not significantly changed during OGD

**Fig. 4:**
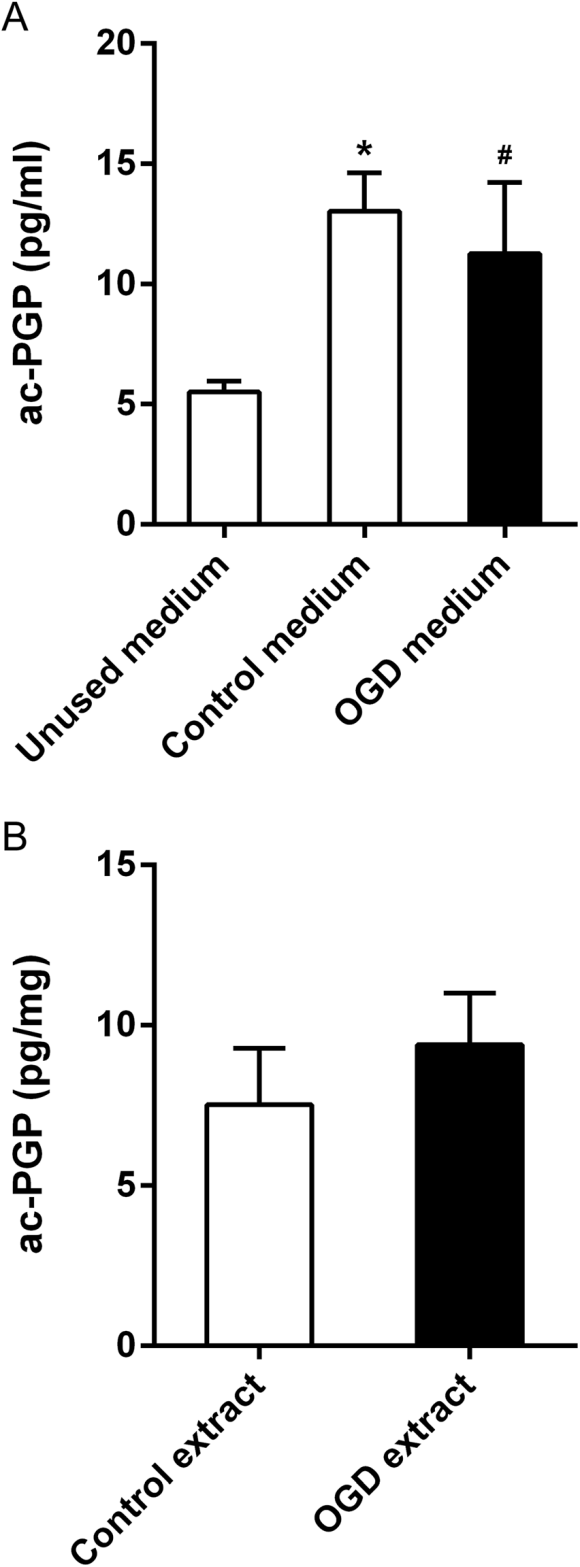
Detection of ac-PGP in culture medium and neuronal cell extracts after OGD. Ac-PGP was detected by LC-MS in **a** unused, control, and OGD culture medium and **b** whole-cell extracts of control cells or cells after 2-h OGD and 24-h reoxygenation. Ac-PGP was significantly increased in control (*p < 0.005) and OGD (^#^ *p* < 0.05) culture medium compared to unused culture medium

Analysis of cell culture medium and cell extracts showed that ac-PGP is both present at low levels in unused cell culture medium and produced at significant levels in neuronal cultures (Fig. 4). Following OGD, no significant changes were observed in the level of ac-PGP in cell culture medium or cell extracts compared to controls (Fig. 4).

### Ac-PGP is significantly upregulated in brain after ischemic stroke

**Fig. 5:**
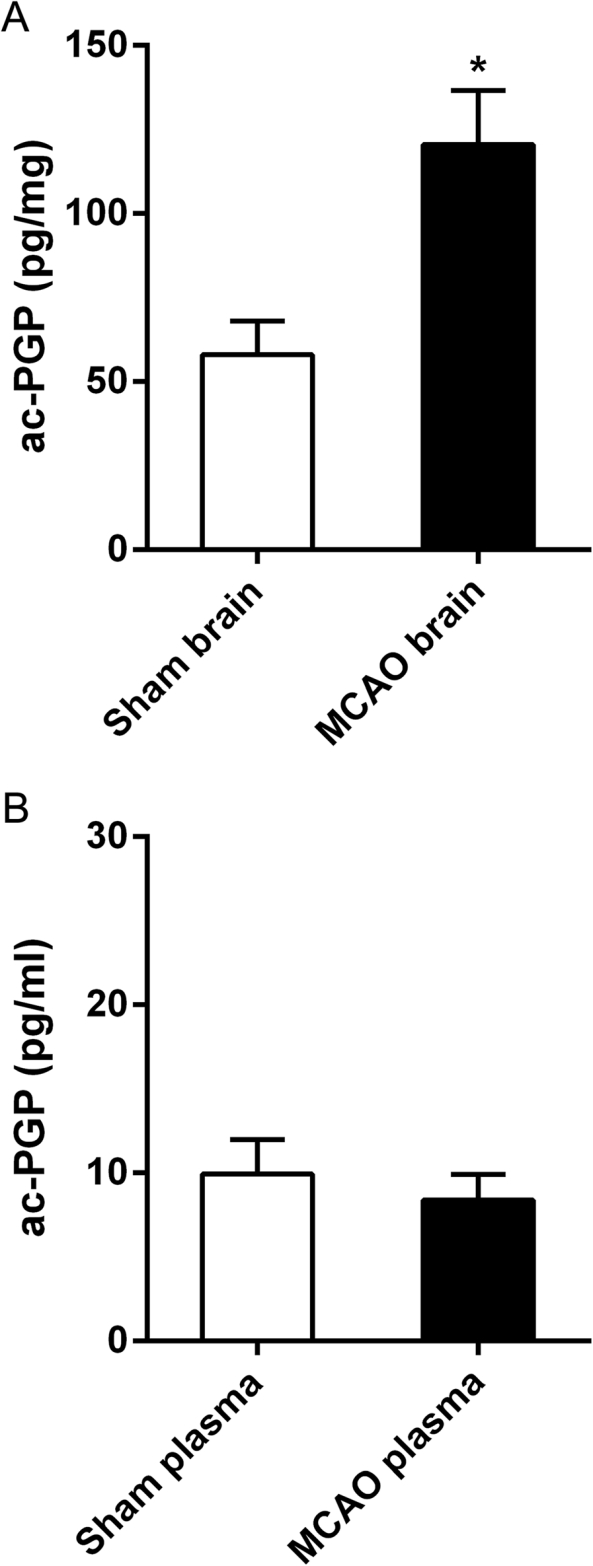
Detection of ac-PGP in rat brain and plasma after stroke. Ac-PGP was detected by LC-MS in **a** brain extracts and **b** plasma from animals undergoing 90-min MCAO with 24-h reperfusion or sham surgery. Ac-PGP was significantly increased in brain in MCAO animals (*p < 0.0001, *n* = 5 animals for each group)

Twenty-four hours after stroke, ac-PGP was significantly upregulated in infarcted cortex compared to sham-operated animals (Fig. 5). Ac-PGP in plasma was not significantly changed in MCAO animals (Fig. 5).

## Discussion

Evidence from several studies suggests a role for CXCR2 receptors in brain neutrophilia and evolution of infarct volume after ischemic stroke. Studies of the CXCR1/2 inhibitor reparixin showed that receptor blockade decreased infarct volume, reduced neutrophil infiltration, and improved long-term neurological outcome in rat models of transient and permanent ischemia [2, 3]. In another study, the CXCR1/2 antagonist G31P significantly decreased infarction when administered after transient MCAO in rats [4]. Further, CXCR2-binding pro-inflammatory chemokines such as CXCL1 and IL-8 are markedly increased in brain and cerebrospinal fluid in inflammatory neurodegenerative diseases, including Alzheimer’s disease, Parkinson’s disease, multiple sclerosis, and stroke, and have been proposed to be mediators of neuronal death, primarily through pro-inflammatory signaling [16–19]. Mounting evidence from in vitro studies, including the results presented in this study, suggest that ligand binding by neuronal CXCR2 receptors mediates neuronal cell death directly, in addition to promoting inflammation which may potentiate long-term neurological damage.

A recent study demonstrated that binding of a rodent IL-8 functional homolog, macrophage inflammatory protein-2 (MIP-2), by CXCR2 receptors induced motor neuron death in primary cultures [20]. In another study, treatment of cultured neurons with IL-8 resulted in the induction of MMP-2 and MMP-9 expression and activities, upregulation of pro-apoptotic proteins, and expression of cyclin D1 [21]. These findings suggest that inflammatory chemokines such as IL-8 and its rodent functional homologs may have direct pathogenic effects in central nervous system disorders independent of their roles in the induction of leukocyte migration and activation. Our in vitro results demonstrating the neurotoxicity of ac-PGP confirm the induction of apoptosis by prolonged activation of neuronal CXCR2 receptors. Neuronal apoptosis after exposure to ac-PGP involves cleavage of caspase-3 and activation of ERK1/2 MAP kinase. While activation of neuronal ERK1/2 by growth factors, ischemic preconditioning, and hypothermia may mediate neuroprotection, activation of ERK1/2 kinase by inflammatory factors in ischemic injury has been shown to promote neuronal apoptosis [22]. Interestingly, activation of ERK1/2 was similarly observed in neutrophils upon exposure to ac-PGP, and an inhibitor of the ERK1/2 pathway attenuated the release of MMP-9 by neutrophils stimulated with ac-PGP [12]. In stem cells, ac-PGP activates ERK1/2 MAP kinase and upregulates CXCR1/2 receptor expression [23]. In another study, ac-PGP was shown to induce increased vascular permeability through CXCR2-mediated induction of signaling pathways in endothelial cells [24]. Thus, in ischemia/reperfusion injury, we hypothesize that the production of ac-PGP in brain may promote inflammation and neurodegeneration through upregulation of CXCR1/2 receptors, increased permeability of the blood-brain barrier, recruitment and activation of leukocytes, and direct neuronal injury mediated through prolonged CXCR2 receptor activation.

The presence of ac-PGP in unused culture medium and significant production of ac-PGP during normal culturing of primary neurons may have important implications for neuronal viability and in vitro studies of neuronal responses to neurotoxic stressors, such as OGD. We hypothesize that ac-PGP in unused culture medium may be of FBS origin. A previous study demonstrated high levels of MMP-2 and MMP-9 in conditioned neuronal culture medium which were unchanged following OGD [11]. Thus, ac-PGP is likely produced in neuronal cultures by the action of MMPs on collagens or other substrates, and the lack of a significant increase in ac-PGP in neuronal cultures after OGD is not surprising. The increase in ac-PGP in brain after stroke and lack of significant change in the level of the molecule after OGD in cultured neurons may be a fundamental difference between in vitro and in vivo stroke models.

## Conclusions

Our findings suggest a new paradigm of injury in stroke and potentially other neurological disorders, wherein an initial injury activates MMPs and promotes chronic inflammation and progressive neuronal injury through sustained production of ac-PGP, a pro-inflammatory and neurotoxic matrix-derived chemokine. Our discovery of the neurotoxic effects of ac-PGP and its upregulation in infarcted cortex provides additional mechanistic rationale for the reported therapeutic efficacy of CXCR2 antagonists in inflammatory neurological disorders and warrants further studies to characterize the role of ac-PGP in neurological injury processes in ischemic stroke.

## Abbreviations

ac-PGG: *N*-acetyl proline-glycine-glycine
ac-PGP: *N*-acetyl proline-glycine-proline
ANOVA: analysis of variance
CF: cystic fibrosis
DAPI: 4',6-diamidino-2-phenylindole
ECM: extracellular matrix
ERK1/2: extracellular signal-regulated kinase 1/2
FBS: fetal bovine serum
GAPDH: glyceraldehyde 3-phosphate dehydrogenase
GRO-α: growth-related oncogene α
GRO-β: growth-related oncogene β
HBSS: Hank’s buffered saline solution
IL-8: interleukin-8
LC-MS: liquid chromatography-mass spectrometry
MAP kinase: mitogen-activated protein kinase
MCAO: middle cerebral artery occlusion
MIP-2: macrophage inflammatory protein-2
MMP: matrix metalloproteinase
OGD: oxygen-glucose deprivation
PBS: phosphate-buffered saline
TTC: 2,3,5-triphenyltetrazolium chloride
